# Rotavirus and *Cryptosporidium* pathogens as etiological proxies of gastroenteritis in some pastoral communities of the Amathole District Municipality, Eastern Cape, South Africa

**DOI:** 10.1186/s13104-020-05024-2

**Published:** 2020-03-30

**Authors:** Luyanda Msolo, Benson C. Iweriebor, Anthony I. Okoh

**Affiliations:** 1 SA-MRC Microbial Water Quality Monitoring Centre, University of Fort Hare, Private Bag X1314, Alice, 5700 Eastern Cape South Africa; 2 Applied and Environmental Microbiology Research Group, Department of Biochemistry and Microbiology, University of Fort Hare, Alice, 5700 Eastern Cape, South Africa; 3 Sefako Makgatho Health Sciences University, Ga-Rankuwa, Pretoria, Gauteng South Africa

**Keywords:** *Rotavirus*, *Cryptosporidium*, Gastroenteritis, Etiology, Enzyme immunoassay

## Abstract

**Objective:**

Cryptosporidium and Rotavirus agents have been associated with severe diarrheal illnesses and remain as one of the worst human health burdens in most developing regions. In the present study, we evaluated the incidences of Cryptosporidium and Rotavirus in diarrheal stool specimens of patients in some rural settlements of the Amathole District Municipality in the Eastern Cape Province, South Africa. Stool specimens from diarrheal children and elderly individuals were collected from clinics and hospitals within the rural communities of the region over a period of 21 months (February 2017–November 2018). Commercial enzyme-immuno-assays were used for the detection of Rotavirus and Cryptosporidium pathogens from processed diarrheal stool specimens.

**Results:**

A total of 53 fresh stool samples from diarrheal patients were screened and 36% of the diarrheagenic stool specimens tested positive for Group A Rotavirus antigens, while 5.7% tested positive for Cryptosporidium antigens. Our findings reveal Rotavirus and Cryptosporidium pathogens as important etiological agents associated with diarrheal illnesses in children, among the rural hinterlands of the Amathole District Municipality.

## Introduction

Several microbial agents have been implicated in diarrheal illnesses and these include bacteria, parasites, and viruses, with the latter being notably the most reported agent over the years [1–4]. Rotaviruses (RoV’s) are the most prevalent etiological agents of severe diarrheal illnesses in children with more than 600,000 deaths reported annually in developing countries [5–9]. The majority of mortalities due to rotavirus infections have reverberated in sub-Saharan Africa, the Indian Sub-continent and Latin Americas [10, 11]. Over 2 million children worldwide are hospitalized because of RoV infections where about 90% of the mortalities are recorded among African and Asian regions each year [12]. Pathogenic parasites remain sixth in the pool of the agents of most detrimental human infectious illnesses, with protozoa being the most common in foodborne epidemics [13]. *Cryptosporidium*, *Giardia,* and *Cyclospora* are pervasive in nature, and have been found in the small intestines and stomach of mammals and are associated with numerous foodborne outbreaks of diarrheal illnesses in developing countries [14, 15]. In sub-Saharan Africa and South Asia, an estimated 2.9 and 4.7 million cryptosporidiosis cases in children less than 2 years old resulted in approximately 202,000 combined annual fatalities, thus accentuating a tremendous health burden in those regions [16]. The epidemiology on the plethora of *Rotavirus* and *Cryptosporidium* infections worldwide is prodigious, however; there are still considerable data gaps in some parts of the world especially the southern parts of South Africa, hence, we evaluated the incidence of *Rotavirus* and *Cryptosporidium* infections among diarrheal patients in some rural settlements in the Amathole District Municipality, Eastern Cape, South Africa.

## Main text

### Methods

#### Study site description

Despite its rural hinterlands, the Amathole District Municipality (ADM) has a relatively high population density of over 880,790 thousand people which is about 12% of the overall population within the province. Significant improvements have been made over the years with respect to water and sanitation in the region, however, about 24% of households in the district still access water from natural water sources including dams, rivers, lakes and household still have significant challenges as far as sanitation is concerned, recording an appallingly high rate of households with no sanitation [17]. This unfortunate state gives a fundamental insight on the high risk of exposure of the local dwellers within these settlements as these natural waters are the most important transmission vehicles of microbes responsible for diarrheal illnesses.

#### Case definition and Sample collection

Diarrheal stool samples were collected from participating patients of all ages who exhibited signs of mild to severe diarrhea and were admitted in both private and public medical facilities situated within or in close proximity to some of the selected rural communities in the ADM of the Eastern Cape Province, and with clear understanding that some patients came from remote areas where medical services and other basic needs such as sanitation and clean water supply were deficient, and had to travel long distances to acquire medical attention.

Samples were collected in sterile screw-capped 50 ml size Falcon tubes and transported in cooler boxes to the Applied and Environmental Microbiology Research Group (AEMREG) laboratory at the University of Fort Hare, for analysis within 6 h of collection. Stool specimens that were fortuitously unsealed in the course of collection and upon arrival to the laboratory before processing were withdrawn from this study.

#### Sample processing

Stool specimens were stored in physiologically buffered saline (PBS) and temporarily stored at 2–8 °C, before the commencement of the assays. The PBS stored samples were used to make 10% dilutions as per the requirements outlined in the Manufacturer’s manual for both Rotavirus and *Cryptosporidium* immunoassay kits.

#### In vitro qualitative immunoassay test for detection of viral and parasitic pathogens

##### ProSpecT™ Rotavirus microplate assay

The commercial ProSpecT™ *Rotavirus* test which utilizes a polyclonal antibody in a solid-phase sandwich enzyme immunoassay to detect group-specific antigen present in Group A rotaviruses was used, following manufacturer’s instructions. The relative sensitivity and specificity of the test is 98.7% and 99.2% respectively.

##### Remel Xpect^®^ Giardia/Cryptosporidium test

The commercial Remel Xpect^®^ Giardia/Cryptosporidium test is a qualitative chromatographic immunoassay that detects the presence of parasitic *Giardia* and *Cryptosporidium* antigens in preserved and unpreserved fecal specimens. This test is rapid and simple to use, with the specificity of about 99% and sensitivity of about 96% and the results are easy to interpret and obtainable within 15 min if carried out correctly.

### Results

#### Detection of Rotavirus antigens

Stool specimens that retained the intense color after the addition of wash buffer were indicative of the presence of these *Rotaviru*s antigens and the absorbance of the positive stool specimens recorded ranged from a minimum of 0.323 to a maximum of 2.906 absorbance units.

A total of 19 (36%) of the tested diarrheagenic stool samples (53) were positive for the Group A Rotavirus. Among the 19 positive specimens, 4 (8%) were obtained from adults and 15 (28%) specimens were recovered from children less than 6 years, thus confirming the predominance of *Rotavirus* infections in pediatrics.

#### Detection of *Cryptosporidium* antigens

Three (5.7%) diarrheagenic stool samples from 53 diarrheal patients were positive for *Cryptosporidium* antigens and this suggests an infection by this parasite, which has been implicated as one of the causes of gastroenteritis [18]. None of the stool specimens tested positive for *Giardia*.

### Discussion

Despite improvements on the development of *Rotavirus* vaccines over the years, which benefit mostly the privileged countries and leaving the less fortunate in despair, Rotavirus in these regions remains one of the peak causes of childhood diarrhea, with an estimated 2.3 million hospitalizations and nearly 527,000 mortalities in children every year especially in destitute countries [19]. The global disease burden and the socio-economic stress brought upon families and the society at large by these Rotavirus infections can never be overemphasized, hence the use of rapid immune-assay for surveillance of these infections is necessary, so as to halt its epidemics [20, 21].

In a similar study by Omoruyi et al. [22], the degree of detection of Cryptosporidium parasites among HIV-patients with diarrhea using the immunoassay methods, was considerably high when compared to Ziehl-Neelsen (ZN) staining methods and surprisingly; Polymerase Chain Reaction (PCR) methods. This revelation also accentuates the importance of chromatographic immunoassays in the screening of clinical samples.

The majority of positive patients were children less than 6 years of age; four patients were adults of which two were diabetic (Table 1). Co-Infection was observed from 2 (3.8%) patients (MHC 03 and KH 04) which may pose an exacerbated diarrheal condition. Prevalence of *Cryptosporidium* among diarrheal specimen collected from a pediatric hospital in Iran has been previously reported [23], which corroborates the findings of our present study.

**Table 1:** Demographic data of the patients who tested positive for *Cryptosporidium* and Rotavirus pathogens

Patient(s)	Age range (years) ≤ 5/6–40/≥ 41	Type of locality (rural/semi-rural/peri-urban/urban)	Source of income (employment)	Total no. of individuals per household	Source of water/storage	Type of sanitary system (toilets/restrooms/lavatories)	Distance to the nearest medical centre/clinic/Hospital (km)	History of diarrhea within the household and period of diagnosis	Immune/health status
P04	≥ 41	Rural	Social grant	8	Tap/storage tanks	Flushable toilets	≈ 5	Yes/3 days	Diabetic
P01	≥ 41	Rural	Social grant	8	Tap/storage tanks	Flushable toilet	≈ 5	No	Diabetic
P03	≤ 5	Rural	–	3	Tap	Pit toilet	≈ 8	No	Healthy
P 07	6–40	Rural	Social grant	6	Tap and storage tanks	Flushable toilet	≈ 12	No	Undisclosed
KH01	≤ 5	Rural	–	11	Tap	Flushable toilets	4	No	Healthy
KH03	≤ 5	Rural	–	4	Tap	Flushable toilets	≈ 8	No	Healthy
KH 04	≤ 5	Rural	–	8	Tap	Flushable toilets	≈ 4	No	Healthy
KH 05	≤ 5	Peri-urban	–	7	Tap	In-house restroom	≈ 1	No	Healthy
KH 07	≤ 5	Rural	–	6	Tap	Flushable toilets	≈ 3	No	Healthy
KH 10	≤ 5	Rural	Social grant	4	Tap	Pit toilet	≈ 8	No	Undisclosed
KH 12	≤ 5	Rural	–	9	Tap	Flushable toilets	≈ 8	No	Healthy
KH 13	6–40	Semi-rural	–	10	Tap/storage tanks	Flushable toilet	≈ 4	No	Undisclosed
MHC 02	≤ 5	Rural	Social grant	8	Tap/storage tank	In-house restroom	≈ 1	No	–
MHC 03	≤ 5	Rural	Social grant	8	Tap	Pit toilet	≈ 7	Yes/2 days	–
MHC 04	≤ 5	Rural	Social grant	5	Tap	Flushable toilet	≈ 5	No	–
MHC 06	≤ 5	Rural	Social grant	5	Tap/storage tanks	Flushable toilet	≈ 5	Yes/2 days	–
Vic 02	≤ 5	Semi-rural	–	8	Tap/storage tanks	Flushable toilet	≈ 2	No	–
Vic 04	6–40	Semi-rural	–	9	Tap/storage tanks	Flushable toilet	≈ 2	Yes	Healthy
Vic 06	≤ 5	Semi-rural	–	4	Tap	Flushable toilet	≈ 2	No	Healthy
UN 01	6–40	Rural	Unemployed	8	Storage tanks	Pit toilet	≈ 8	No	Undisclosed
R07	≥ 41	Peri-urban	Employed	5	Storage tanks	Flushable toilet	≈ 2	Yes	Undisclosed

Though the immunoassays used in this study have been proven to be expedient, simple, rapid and results obtainable timeously, however, numerous studies have highlighted the necessity for further molecular investigations as paramount for extensive and indisputable detection of these pathogens [24]. Nonetheless; previous studies have recommended the utilization of rapid immunoassay tests for timeous and effective therapy [25]. Furthermore, the use of such tests ensures that accuracy and efficiency are maintained throughout the diagnosis of enteric pathogens especially in situations where there is a lack of resources, labor and time [26]. Polymerase Chain Reaction (PCR) techniques continue to champion the diagnosis of *Cryptosporidium* infections owing to their improved sensitivity and specificity, however, these methods are of restricted applicability due to their costs, high technical expertise demand and special equipment required, which then seclude the less fortunate countries from indulging on such techniques [22]. In a study conducted in one of the provinces of South Africa, a prevalence of about 43% of *Cryptosporidium* species was obtained in surface waters, which were primarily used for irrigation and other domestic purposes [27]. These findings highlight the association of this pathogen with environmental waters and subsequently fresh produce, thereby accenting the risk of exposure of humans who utilize these waters. Furthermore, the steadiness of enteric parasites in the environment suggests that open water sources with a high possibility of anthropogenic, wildlife and livestock interactions may exacerbate the risk of cryptosporidiosis of public health importance [28]. The efficacy of Enzyme Immuno Assays (EIA) in the diagnoses of various microbial pathogens is seen by others to be inefficient compared to other methods like Reverse Transcription-Polymerase Chain Reaction (RT-PCR). However, previous studies have complete corroboration between both methods [29], thereby validating the efficiency of the detection method used in this study. Moreover, these techniques are less time consuming with high sensitivity and specificity, making it one of the most valuable rapid methods of detection in vitro. Our findings further corroborate those of another report and are in support of the notion that modern immune-assays serves as a good alternative in diagnoses of clinical infections [30]. Several studies have fallen short in relating rotavirus-mediated diseases with climatic conditions [5] and likewise, this current study could not draw a precise conclusion in relation to climate conditions, since specimens and cases were obtained irrespective of the season nor climate condition. Among the provinces in South Africa, the Eastern Cape is one of the poorest of the 9 provinces; comprising of a number of rural and informal settlements with poor infrastructure and limited or no access to basic services such as health care facilities, proper hygiene and adequate sanitation practices, potable and safe drinking water; leaving the populace in this province at the highest risk of exposure to numerous food and water-borne diseases (including diarrhea), thus constituting a serious public and environmental health burden, and subsequently a severe strain in socio-economic lifestyle in the province.

### Conclusion

*Rotavirus* and *Cryptosporidium* pathogens are gastroenteritis agents capable of causing a series of diarrheal episodes predominantly in children less than six years of age. This revelation is largely demonstrated in regions with less to complete lack of resources and campaigns for serious and insightful concerns about public health. The prevalence of *Rotavirus* and *Cryptosporidium* enteric pathogens highlighted in this study from the rural hinterlands of the Eastern Cape Province justifies the need for cross-sectional and collaborative strategies from the higher authorities to implement as a matter of urgency the provision and distribution of vaccines as to combat the dissemination of these enteric pathogens. There is a need for extension of alms from public health sectors so as to reach even the utmost remote areas where these infections are endemic and providing the critically needed services in order to ameliorate this plight.

## Future prospects

Exploration of more gastroenteritis agents in a larger population is paramount in unwinding and understanding the trends, eco-distribution of these etiologic agents and defining their roles, so as to minimize the therapeutic and public health burden they pose. Furthermore; molecular characterization of these etiological agents is imperative as a means of understanding the mechanisms of action encompassed by these enteric pathogens at a cellular and molecular level.

## Limitations

- One of the outstanding challenges encountered in the course of our study was the reluctance of some patients to partake in the study, by declining or prohibiting us to take samples from them even though they exhibited signs and symptoms or were diagnosed with diarrheal illness. An overall sum of 12 individuals declined to be included in this study even though they presented symptoms of diarrhea.
- This then resulted in the acquisition of fewer than expected specimens, though reports from medical midwives revealed considerable amounts of cases or hospitalizations in the regions under study. Stool specimens that were found unsealed in the course of collection and upon arrival to the Laboratory were withdrawn from this study.
- Also; it became apparent that some individuals fail to seek medical attention even though exhibiting signs of the illness, this is mainly due to the extended distances to health facilities, and hence some cases may have been missed or misdiagnosed.

## Data Availability

Data will be made available on reasonable request.

## Abbreviations

AG: Acute Gastroenteritis
ADM: Amathole District Municipality
AEMREG: Applied and Environmental Microbiology Research Group
AMGDS: Amathole Municipality Growth and Development Summit
HIV: Human immunodeficiency virus
PBS: Physiological buffered saline
EIA: Enzyme immuno assays
PCR: Polymerase chain reaction
RT-PCR: Reverse transcription-polymerase chain reaction
ZN: Ziehl–Neelsen
